# Potential cardiovascular adverse events when phenylephrine is combined with paracetamol: simulation and narrative review

**DOI:** 10.1007/s00228-015-1876-1

**Published:** 2015-05-29

**Authors:** Hartley C. Atkinson, Amanda L. Potts, Brian J. Anderson

**Affiliations:** AFT Pharmaceuticals Ltd, PO Box 33-203, Takapuna Auckland, 0740 New Zealand; Clinical Solutions NZ Ltd, PO Box 10574, Te Rapa Hamilton, 3241 New Zealand; Department of Anaesthesiology, University of Auckland, Private Bag 92019, Auckland, 1142 New Zealand

**Keywords:** Paracetamol, Phenylephrine, Interaction, Pharmacodynamics, Blood pressure

## Abstract

**Background:**

Increased bioavailability of phenylephrine is reported when combined with paracetamol in over-the-counter formulations for the symptomatic treatment of the common cold and influenza. Such formulations could increase phenylephrine-related cardiovascular adverse events particularly in susceptible individuals. Quantification of the effect of phenylephrine concentration on blood pressure allows simulation of potential adverse combination therapy effects.

**Methods:**

MEDLINE and EMBASE databases were searched for papers discussing or describing any adverse effect, hypersensitivity or safety concerns related to phenylephrine alone or in combination with other drugs.

The pharmacodynamic relationship between plasma phenylephrine concentration and mean arterial blood pressure was characterized using published observations of blood pressure changes after ophthalmic eye drops. The resulting pharmacokinetic and pharmacodynamic parameters were then used to predict mean arterial blood pressure (MAP) changes in that population if given an oral combination of phenylephrine and paracetamol.

**Results:**

There were 1172 papers identified for examination. Forty-seven reports fulfilled the inclusion criteria. Increases in blood pressure and decreases in heart rate have been reported with doses over 15 mg. It has been estimated that a 20-mmHg increase in systolic blood pressure would occur with an oral dose of 45 mg phenylephrine in normotensive healthy people. Those taking monoamine oxidase inhibitors report increased systolic blood pressure of greater than 60 mmHg. Blood pressure and heart rate changes are potentiated in patients with underlying hypertension. Simulation showed a modest increase in MAP when phenylephrine 10 mg was co-administered with paracetamol 1 g (4.2 vs 12.3 mmHg).

**Conclusions:**

Combination paracetamol phenylephrine oral therapy has potential to increase blood pressure more than phenylephrine alone in those with cardiovascular compromise.

**Electronic supplementary material:**

The online version of this article (doi:10.1007/s00228-015-1876-1) contains supplementary material, which is available to authorized users.

## Introduction

Phenylephrine is a selective alpha-1 adrenoceptor agonist with powerful vasoconstrictive properties. Historically, its use had been restricted to the perioperative period and intensive care medicine for preparation of the surgical field and control of haemorrhage during ear nose and throat procedures, pupillary dilation and maintenance of blood pressure. Phenylephrine is commonly used now as a nasal decongestant in many over-the-counter (OTC) cold and influenza preparations.

When phenylephrine was combined with another commonly administered cold and flu medication, paracetamol, the plasma concentration of phenylephrine was, on average, twice that obtained when phenylephrine was given alone and the peak concentration approximately four times higher [1]. It is suggested that the increase in phenylephrine bioavailability is due to a reduction in the amount of phenylephrine undergoing first-pass metabolism due to saturation of the sulfation pathways by paracetamol [1]. Formulation may also have impact. Phenylephrine bioavailability was reduced when administered as a paracetamol-guaifenesin-phenylephrine syrup compared to the same combination in tablet form [2]. These findings raise the concern that phenylephrine administered as a combination with paracetamol may increase the incidence of adverse effects attributable to phenylephrine, most notably cardiovascular adverse effects particularly those with preexisting cardiovascular conditions.

Here, we provide a systematic narrative review of cardiovascular adverse effects associated with phenylephrine. We quantify the effect of phenylephrine concentration on blood pressure using published data and simulate the potential impact paracetamol phenylephrine combination oral therapy may have on cardiovascular endpoints.

## Methods

### Literature review of phenylephrine adverse events

#### Search strategy

A broad search (search terms are detailed in the Supplementary Appendix) of both MEDLINE and EMBASE databases was undertaken followed by manual selection of relevant reports based on the inclusion and exclusion criteria described below. No specific time limits were applied to the search. The time frame of the search was limited only by the coverage of the database (MEDLINE: 1946 to April 2014; EMBASE: 1947 to April 2014).

Papers discussing or describing any adverse effect, hypersensitivity or safety concern related to phenylephrine alone or in combination were included. Papers were excluded if they (1) did not describe an adverse effect for phenylephrine; (2) related to children under 12 years of age; (3) were not written in English or a full-text version was not available for purchase; (4) was not a clinical trial [either prospective or retrospective], case report or series, or a meta-analysis; and (5) did not relate to the cardiovascular system. References of identified papers were reviewed for additional relevant reports.

### Simulation

#### Phenylephrine PKPD relationship analysis

The relationship between plasma phenylephrine concentration and mean arterial blood pressure was characterized using those published data from Kumar and colleagues [3], who related the systemic absorption of phenylephrine eye drops to cardiovascular effects. Individual plasma concentrations and corresponding blood pressure changes at 0, 10, 20 and 60 min after 2.5 % (*n* = 10) and 10 % (*n* = 10) eye drops (two 32-μL drops at 5-min intervals) are contained in Tables 1 and 2 of that publication(*n* = 20). Further pharmacokinetic (PK) data were available from healthy volunteers given oral phenylephrine 10 mg alone, with blood for concentration assay taken at 5, 15, 30, and 45 min and 1, 2, 3, and 6 h (*n* = 28, data from [1, 4]). Intravenous time-concentration data were available from a study by Hengstmann and colleagues [5]. Four healthy volunteers were given phenylephrine 1 mg, and blood was taken for assay on 17 occasions over the subsequent 4 h. Pooled data for that study are presented in Table 2 of that publication(*n* = 1). Technical methods for population parameter estimates using nonlinear mixed effects models (NONMEM) can be found in the Supplementary Appendix.

**Table 1:** Cardiovascular effects following administration of phenylephrine alone or in combination with other medications—overview of literature review

Phenylephrine dose	Population	Result	Comments	Reference
Oral				
10—25 mg	Normotensive participants with common cold (*n* = 48)	↑ HR cf. placebo doses ≥10 mg ↓ BP cf. placebo doses ≥15 mg		Cohen et al. [6]
10 mg	Normotensive participants with nasal congestion (*n* = 88)	NC BP	Conflicting results —20 % of participants showed ↑ BP, while 32 % showed ↓ BP 60 min after administration —30 % of participants showed ↑ HR, while 44 % showed ↓ HR 60 min after administration —Nine participants had ↑HR >10 bpm	McLaurin et al. [7]
250 mg	Normotensive volunteers (*n* = 7)	Average ↓ in HR = 21 bpm Average ↑ in BP = 31 mmHg	Threshold for pressor response estimated to be 50 mg.	Keys and Violante [8]
45 mg	Normotensive volunteers (*n* = 4), concurrent MOA inhibitors	Significant ↑ in BP Significant ↓ in HR	≥67 mmHg ↑ in BP in two participants administered single 45-mg dose of phenylephrine after 7 days of MOA inhibitor therapy (tranylcypromine or phenelzine). Required intervention with phentolamine.	Elis et al. [9]
10 mg	Normotensive volunteers (*n* = 4), concurrent MOA inhibitors	Significant ↑ in BP	20 mmHg ↑ in BP after 10 mg phenylephrine dose following 11 days on tranylcypromine	Elis et al. [9]
Unclear—?10 mg	74-year-old women with comorbidities, concurrent MOA inhibitors	Significant ↑ in BP (hypertensive crisis—BP up to 230/120 mmHg)	Combination of phenylephrine from an over-the-counter preparation, terbutaline and taloxatone.	Lefebvre et al. [10]
Unclear—overdose	24-year-old otherwise healthy male	Significant ↑ in BP (196/100 mmHg, ↓ in HR (40 bpm), third-degree atrioventricular block	Ingestion of 30–40 tablets each containing 40 mg phenylpropanolamine, 10 mg phenylephrine, 5 mg chlorpheniramine and 15 mg phenyltoloxamine	Burton et al. [46]
Unclear—up to 60 mg/day for 4 days	59-year-old otherwise healthy female	ICH, SAH	Took cold and flu medications containing PE, paracetamol, dextromethorphan and chlorpheniramine for 4 days before hospitalization	Tark et al. [36]
Topical (eye drops, nasal packs, nasal sprays)				
2.5 or 10 % single eye drop	Cataract surgery—normotensive and hypertensive patients (*n* = 89)	↑ BP following both 2.5 and 10 % eye drop in both normotensive and hypertensive patients.	4/30 patients in 10 % group and 1/29 patients in the 2.5 % group developed severe hypertension which required intravenous hypotensive agents for management—all patients who developed severe hypertension had baseline hypertension.	Chin et al. [11]
2.5 or 10 % single eye drop	Cataract surgery—normotensive and hypertensive patients (*n* = 49)	↑ BP following both 2.5 and 10 % eye drop in both normotensive and hypertensive patients.		Kenawy et al. [47]
2.5 % eye single drop	Cataract surgery—normotensive and hypertensive patients (*n* = 217)	NC BP NC HR	Severe hypertension, unstable angina and uncontrolled diabetes all exclusions. Cardiovascular endpoints measured at beginning and end of procedure—end time not defined—not continuously monitored	Lam et al. [48]
2.5 or 10 % single eye drop	Cataract surgery—normotensive (*n* = 54)	NC BP NC HR	Normotensive participants only—no history of cardiovascular disease	Malhotra et al. [49]
2.5 or 10 % single eye drop	Funduscopy (*n* = 29)	NC BP NC HR	Normotensive participants only	Motta et al. [50]
10 % single eye drop	49-year-old female, medically controlled hypertension	SAH, rupture cerebral aneurysm, BP 200/108 mmHg, HR 48 bpm, death	Possible interaction with propranolol—not able to vasodilate to counteract pressor effect of PE	Cass et al. [40]
2.5 % single eye drop	72-year-old female, medically controlled hypertension, diabetes mellitus	ICH, BP 300/200 mmHg		Weisberg et al. [41]
10 % four times eye drops	57-year-old man, no known cardiovascular disease	Acute myocardial infarct, severe hypertension and cardiac arrhythmia		Lai [51]
500 mg via nasal pack	30-year-old man, healthy	Severe hypertension (210/146 mmHg) and bradycardia (45 bpm)		Macmillan and Barker [52]
0.25 % solution via nasal pack	23-year-old woman, no coronary artery disease	Myocardial infarct	Combined with 4 % cocaine for local anaesthesia	Ashchi et al. [13]
0.25 % solution via nasal pack	28-year-old man	Acute hypertension (190/100 mmHg), pulmonary oedema	Patient had history of smoking cocaine	Singh et al. [12]
Unclear—?daily nasal spray TID	57-year-old male	ICH, SAH, occipital infarct, BP 240/110 mmHg	Nasal spray TID for 4 months	Cantu et al. [37]
Unclear—nasal spray	30-year-old, postpartum female	SAH	Nasal spray daily for 1 week	Chartier et al. [38]
Unclear—nasal spray	45-year-old female	SAH	Nasal spray for many weeks	Genonceaux et al. [39]
Injectable				
25 μg/min 50 μg/min 100 μg/min intrathecal infusion	Women scheduled for elective caesarean section (*n* = 75)	Significant reduction (up to 20 %) in HR and CO in 100 μg/min group		Stewart et al. [53]
100 μg/min intrathecal infusion	31-year-old woman, emergency caesarean section	Ventricular bigeminy		Lai et al. [54]
150 μg iv	35-year-old woman, elective caesarean section	ICH	Three 50-μg doses of PE for treatment of hypotension	Ranasinghe et al. [42]

↑ increase, ↓ decrease, *cf*. compared with, *NC* no change, *HR* heart rate, *BP* blood pressure, *PE* phenylephrine, *CO* cardiac output, *TID* three times daily, *QID* four times daily, *ICH* intracerebral haemorrhage, *SAH* subarachnoid haemorrhage

**Table 2:** Standardised phenylephrine population pharmacokinetic and pharmacodynamic parameter estimates for ophthalmic PKPD analysis

Parameter		Estimate	%BSV	95 % CI
Pharmacokinetics				
CLstd (L h^−1^ 70 kg^−1^)		139	38.3	61.5, 235.9
V1std (L 70 kg^−1^)		15.3	106.3	9.5, 73.9
Qstd (L h^−1^ 70 kg^−1^)		30	130	23.9, 311
V2std (L 70 kg^−1^)		235	101	102, 562
Tabs oral (h)		0.52	40	0.34, 0.63
Lag oral (h)		0.247	–	0.21, 0.26
Bioavailability oral		0.014		0.008, 0.051
Tabs ophthalmic 2.5 % (h)		0.07	71	0.03, 0.11
Bioavailability 2.5 % ophthalmic solution		0.15		0.077, 0.506
Tabs ophthalmic 10 % (h)		0.275	–	0.21, 0.34
Bioavailability 10 % ophthalmic solution		0.14		0.071, 0.557
Residual error	Additive (mcg L^−1^)	0.013	–	0.001, 0.015
	Proportional (%)	32.6		25.2, 39.9
Pharmacodynamics				
E0 (mmHg)		86.3	4.4	81.6, 90.2
EC50 (mcg L^−1^)		11.1	141	1.67. 19.9
Emax (mmHg)		51.2	44.8	20.5, 109.1
Residual error	Additive (mcg L^−1^)	9.88	–	7.03, 11.7
	Proportional (%)	61.6	–	0.6, 125

*BSV* between-subject parameter variability, *CI* confidence interval

## Results

### Literature search

A total of 1172 papers were identified for examination. Forty-seven reports fulfilled the inclusion criteria. The majority of literature concerning phenylephrine and cardiovascular effects related to its use as a hypertensive agent for the management of hypotension associated with shock and spinal anaesthesia. These effects are therapeutic in these scenarios, and as they are not adverse effects, they are not discussed. Case reports and studies that described unexpected or unwanted cardiovascular effects following the use of phenylephrine are listed in Table 1.

The standard OTC 10-mg dose of phenylephrine appears to be well tolerated by the majority of people; however, increases in blood pressure and decreases in heart rate are reported with doses over 15 mg [6, 7]. It has been estimated that a 20-mmHg increase in systolic blood pressure would occur with an oral dose of 45 mg phenylephrine in normotensive healthy people [8]. This situation changes considerably in people taking medications such as monoamine oxidase inhibitors where interaction with phenylephrine caused increases in systolic blood pressure of greater than 60 mmHg and required intervention [9, 10]. Blood pressure and heart rate changes also appear to be potentiated in patients with underlying hypertension. One study reports severe hypertensive episode requiring intervention in 10 % of study participants given 10 % topical drops as a mydriatic agent in ophthalmic surgery, all of whom had underlying hypertension; no episodes of hypertension were reported in normotensive participants [11]. Phenylephrine may also interact with cocaine (medical or recreational) potentiating the hypertensive effects of phenylephrine [12, 13].

Unwanted cardiovascular effects are commonly reported when phenylephrine is administered intravenously for its hypertensive effects and appear to be dose dependent [14–19]. The majority of literature relates to bradycardia and reactive hypertension when phenylephrine was used to counter the hypotensive effects of spinal anaesthesia during caesarean section [14–19]. An increase in blood pressure with associated impairment in myocardial perfusion was seen when phenylephrine was administered to patients with underlying cardiac disease (angina pectoris, old myocardial infarct or chronic coronary artery disease) [20]. Increased blood pressure [21–27], vasoconstriction resulting in worsening of orthostatic intolerance [28], atrial fibrillation after coronary artery bypass surgery [29], decreased cerebral oxygenation [30, 31], bradycardia in patients with high cervical spinal cord injury [32], cardiac arrhythmias [33], pulmonary oedema and myocardial infarction [34], and microvascular occlusion syndrome [35] have all been associated with phenylephrine use.

Cerebrovascular events have also been reported. Tark et al. report the case of an otherwise healthy 50-year-old woman who suffered intracerebral haemorrhage after oral administration of standard doses of cold medicines containing phenylephrine and paracetamol for 4 days before hospitalization [36]. Other studies have reported cerebrovascular accidents following phenylephrine use via topical and intravenous administration [37–42].

### PKPD relationship analysis

**Fig. 1:**
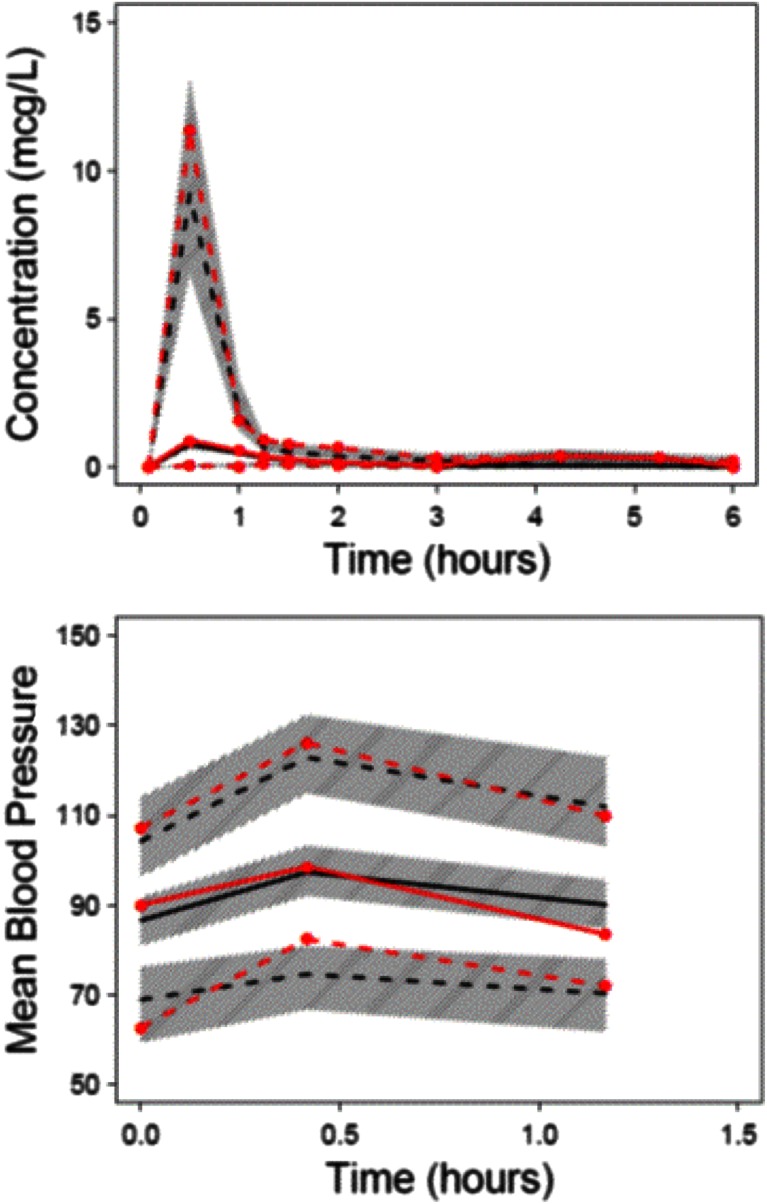
Visual predictive check for the PKPD model. All plots show median and 90 % intervals (*solid* and *dashed lines*). Also shown are prediction percentiles (10, 50 and 90 %) for observations (*lines with symbols*) and predictions (*lines*) with 95 % confidence intervals for prediction percentiles (*grey*-*shaded areas*). The *upper panel* is the pharmacokinetic fit using PK data from all formulations while the *lower panel* is the pharmacodynamic fit involving data only from subjects undergoing ophthalmic surgery

**Fig. 2:**
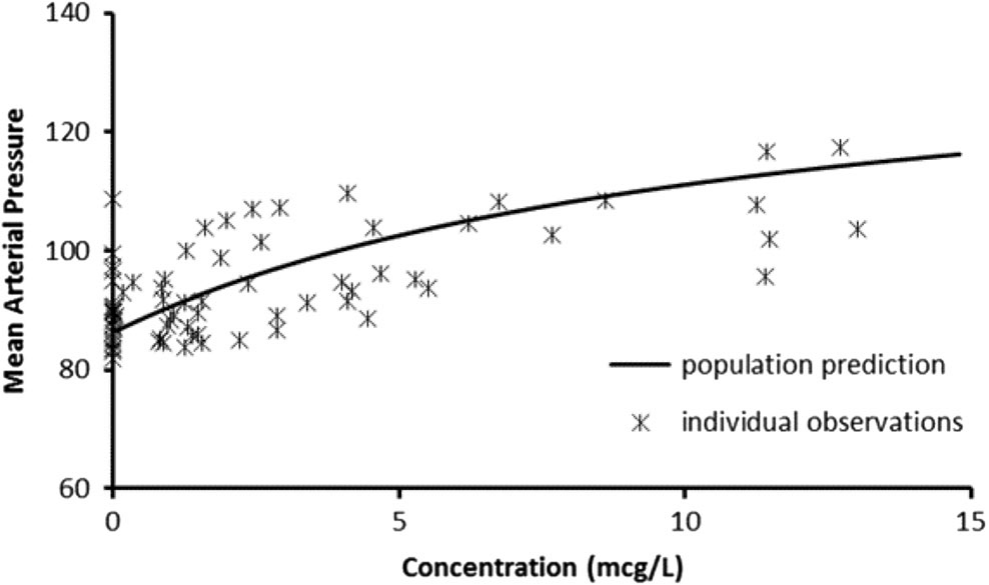
The relationship between phenylephrine concentration and MAP in patients undergoing ophthalmic surgery

The pharmacokinetic/pharmacodynamic (PKPD) analysis was based on 49 subjects (with 387 observations) who received phenylephrine ophthalmic eye drops where plasma concentrations were available for analysis. Patients had a mean age 34.3 SD 8.2 years and a mean weight 74.4 SD 3.4 kg. Pharmacokinetic and pharmacodynamic parameter estimates are reported in Table 2. The correlation of between parameter variability is shown in Table 3. PC-VPC plots, used to demonstrate goodness of fit, are shown in Fig. 1. Figure 2 demonstrates the pharmacodynamic relationship between phenylephrine concentration and MAP for a typical individual.

**Table 3:** The correlation of parameter between-subject variability

	CL	Q	V1	V2	Tabs
CL	1				
Q	−0.799	1			
V1	−0.817	0.651	1		
V2	−0.630	0.093	0.533	1	
Tabs	0.512	−0.463	−0.148	−0.239	1

### Simulation

**Fig. 3:**
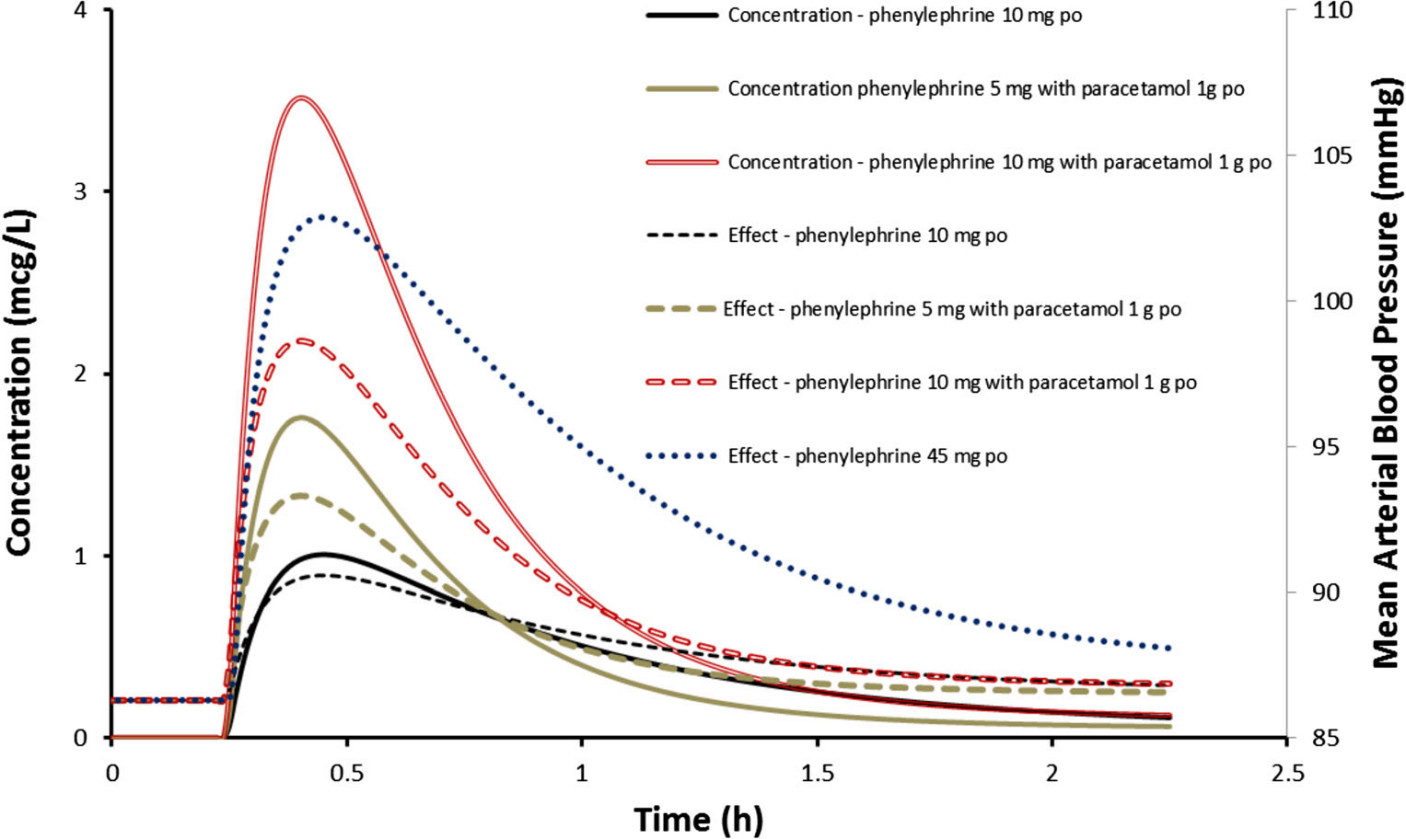
Pharmacodynamic parameter estimates from patients (mean age 34.3 years, 70 kg) undergoing ophthalmic surgery were combined with derived pharmacokinetic estimates from the current study to simulate mean time-concentration (*solid lines*) and mean arterial blood pressure changes (*dashed lines*) that might occur when given oral phenylephrine with and without paracetamol

Pharmacodynamic parameter estimates estimated from the phenylephrine ophthalmic study [3] were combined with derived pharmacokinetic estimates from a study in healthy volunteers given paracetamol and phenylephrine combination therapy [4] to simulate mean time-concentration and mean arterial blood pressure changes that might occur if patients were given oral phenylephrine with and without paracetamol. These simulations were performed using Berkeley Madonna™ modelling and analysis of dynamic systems software V 8.3.18 (Robert Macey and George Oster of the University of California, Berkeley, USA). We predict an increase in MAP of 16 mmHg after 45 mg phenylephrine; i.e., a person with a BP of 120/65 mmHg might increase to 140/80 mmHg, a systolic increase of 20 mmHg. Plots are presented in Fig. 3. The increased absorption rate of phenylephrine when combined with paracetamol results in higher peak concentrations than might be anticipated from increased bioavailability alone.

## Discussion

Phenylephrine has now replaced pseudoephedrine in most over-the-counter (OTC) cold and influenza medications. There are few data reporting adverse events associated with oral phenylephrine use. What little information available must be gleaned from other routes of administration where more formal studies have been conducted: phenylephrine interacts with monoamine oxidase inhibitors and possibly other drugs to potentiate its hypertensive effect; cardiovascular changes may be more pronounced in people with underlying cardiovascular disease and may lead to decreased myocardial oxygenation, cardiac arrhythmias, decreased cerebral oxygenation and exaggerated vasoconstriction and stroke.

That few adverse events following oral administration of phenylephrine are reported is not surprising, though not necessarily reflective of the actual incidence of adverse effects. Relative oral bioavailability remains poorly documented but may be as little as 0.003 [5]. Absorption is slow (Tabs 0.4 h, BSV 30.8 %), and peak concentrations will be less than that observed after rapid intravenous administration. Oral phenylephrine is generally administered in a community setting to relieve symptoms of malaise associated with colds and influenza, and as such, blood pressure changes over the short duration of phenylephrine administration are unlikely to be recorded. The few studies examining oral phenylephrine at the recommended dose of 10 mg have shown it to be well tolerated in patients suffering from nasal congestion. However, these studies focus on phenylephrine as a single agent and not in combination with paracetamol where bioavailability is increased and peak plasma concentrations doubled [1]. Furthermore, these studies have primarily been conducted in either healthy volunteers or in otherwise healthy patients with nasal congestion.

The simulation study assumes that the administration of phenylephrine with paracetamol more than doubles the bioavailability of phenylephrine and reduces the absorption half-time by 50 % resulting in a doubling of phenylephrine plasma concentration and an approximate fourfold increase in Cmax, with large between-subject variability [4, 1]. Of concern is the possibility of increased adverse effects associated with this increase in plasma concentration, particularly in people with cardiovascular compromise or on other medications that may interact with phenylephrine. Simulation using blood pressure changes after ophthalmic administration provides an example of the magnitude of blood pressure change for a typical subject: a standard 10-mg dose of phenylephrine combined with paracetamol could result in an increase in MAP of more than 10 mmHg (Fig. 2). We report considerable between-subject variability that was unexplainable from the limited cohort investigated. The impact of age, existing hypertension and ophthalmic preparation dose accuracy are covariates that require further investigation.

An important consideration is the substantial variability in Cmax [4]: a subgroup of the population would be exposed to relatively higher phenylephrine plasma concentrations than the population mean data would suggest, leading to more serious adverse events. Indeed, one case report describes haemorrhagic stroke in an otherwise healthy female taking phenylephrine and paracetamol in combination for treatment of cold and flu symptoms [36]. It is also possible that others without underlying hypertension could also be compromised. It has been estimated that a 20-mmHg increase in blood pressure would occur with a 45-mg phenylephrine dose in normotensive patients [8], an observation consistent with our current simulation. It is possible that any given individual may experience a plasma concentration similar to that seen with a 45-mg dose when 10 mg phenylephrine is combined with paracetamol. Systemic or pulmonary hypertension is also reported in children (6 months–14 years) administered 10 % ophthalmic drops [43].

When medications are administered OTC, the burden of safety should be high—with only 40 % of consumers reading the packet label when they take a medicine for the first time and only 7 % reading safety information and warnings [44], and changing labelling to reduce the risk of adverse events is ineffective. In addition, more than one third of consumers take more than the recommended dose of an OTC medication believing that it will increase the effectiveness of the medication and an additional 30 % combine different OTC medicines to treat different symptoms [44] leading to the potential for overdose of any single active ingredient and the associated adverse effects.

In 2007, the FDA determined that there was insufficient data to support increasing the dose to the higher 25 mg whilst maintaining a similar safety profile to the 10-mg dose [45]. At the same time, the committee concluded that “comparisons of the pharmacokinetics of single-ingredient products versus multiple ingredient products” and “safety evaluations of the effects of phenylephrine on blood pressure and cardiovasculature and use of phenylephrine in patients with important comorbidities such as BPH, hypertension or diabetes mellitus” be conducted [45]. Whilst there are now some data on the interaction pharmacokinetics of phenylephrine, the consequences to the population of the OTC co-administration of phenylephrine and paracetamol remain difficult to define. The literature reviewed here suggests that this increased phenylephrine exposure could be associated with many, potentially life-threatening adverse events in individuals both with underlying cardiovascular compromise and those who are otherwise healthy. Despite the FDA’s call for studies almost a decade ago, there remain no studies specifically examining the safety profile of phenylephrine in populations with underlying compromise, nor are there any studies examining the effects of chronic use of phenylephrine despite the potential safety burden.

## Electronic supplementary material

ESM 1 (DOCX 26 kb)
