# Supplementary material for: Potential cardiovascular adverse events when phenylephrine is combined with paracetamol: simulation and narrative review
Source: Eur J Clin Pharmacol. 2015 May 29;71(8):931–8. doi: 10.1007/s00228-015-1876-1 (PMC4500855; doi:10.1007/s00228-015-1876-1)
Supplement: Supplementary file 1 — (DOCX 26 kb) [file 228_2015_1876_MOESM1_ESM.docx]

**Potential Cardiovascular Adverse Events when Phenylephrine is combined with Paracetamol: Simulation and Narrative Review.**

**Supplementary Appendix**

**Supplementary Methods**

**Literature Review Search Terms**

MEDLINE (via OVIDSP on 10/03/2015)

| **Search #** | **Search Terms** | **Results** |
| --- | --- | --- |
| 1 | (adolescent/ or adult/ or middle aged/ or aged/) not child/ | 5258456 |
| 2 | human/ or male/ or female/ | 15542248 |
| 3 | comparative study/ or randomized controlled trial/ or prospective study/ or retrospective study/ or controlled study/ or meta analysis/ or case report/ or case series/ | 4350345 |
| 4 | phenylephrine/tu or phenylephrine/ch or phenylephrine/du or phenylephrine/ae or phenylephrine/po or phenylephrine/pd or phenylephrine/ad | 11455 |
| 5 | phenylephrine.mp. and (drug therapy/ or drug combinations/ or drug therapy, combination/) | 431 |
| 6 | phenylephrine.ti. | 1657 |
| 7 | 4 or 5 or 6 | 11811 |
| 8 | 1 and 2 and 3 and 7 | 942 |

###### EMBASE (via OVIDSP on 10/03/2015

| **Search #** | **Search Terms** | **Results** |
| --- | --- | --- |
| 1 | adolescent/ or adult/ or aged/ | 5840703 |
| 2 | Human/ or male/ or female/ | 16970430 |
| 3 | comparative study/ or randomized controlled trial/ or retrospective study/ or prospective study/ or controlled study/ or systematic review/ or meta analysis/ | 5634088 |
| 4 | phenylephrine/ | 27698 |
| 5 | phenylephrine.ti. | 1875 |
| 6 | 1 and 2 and 3 and 4 and 5 | 230 |

**Simulation**

**Phenylephrine PKPD Relationship Analysis**

Population parameter estimates were obtained using non-linear mixed effects modeling (NONMEM 7.3, Globomax LLC, Hanover, MD, USA). This software accounts for population parameter variability (between subjects) and residual variability (random effects) as well as parameter differences predicted by covariates (fixed effects). The population parameter variability (or between subject variability, BSV) in structural model parameters was modelled by exponentiating random effects (equivalent to assuming a log-normal distribution). Residual unknown variability was modelled using additive and proportional terms. A first order absorption, first order elimination two-compartment model was parameterized in terms of an absorption half-time (Tabs), central and peripheral volumes (V1, V2), inter-compartment clearance (Q) and clearance (CL).

A lag time (Tlag) before absorption for each formulation was investigated. Absorption half-time for each enteral formulation (oral, ophthalmic 2.5%, ophthalmic 10%) was calculated from the absorption rate constant (Ka)

$$Tabs=\frac{{log}_{e}2}{Ka}$$

Bioavailability relative to the intravenous formulation was also estimated. The first order conditional estimation with interaction estimation method with differential equations using ADVAN4 TRAN4 was used to estimate population mean parameters, between subject variance and residual variance. Convergence criterion was 3 significant digits.

The population parameter variability was modelled in terms of random effect (η) variables. Each of these variables was assumed to have mean 0 and a variance denoted by ω2, which was estimated.

The covariance between two elements of η (e.g. CL and V) is a measure of statistical association between these two variables. Their covariance is related to their correlation (R) i.e.

R = covariance/√ (ω2CL x ω2V)

The parameter values were standardised for a body weight of 70 kg using an allometric model [1,2].

$$Pi=Pstd\cdot\left( \frac{Wi}{Wstd} \right)^{PWR}$$

where Pi is the parameter in the ith individual, Wi is the weight in the ith individual and Pstd (e.g., CLstd, Qstd, V1std, V2std) is the parameter in an individual with a weight Wstd of 70 kg. The PWR exponent was 0.75 for clearance, 0.25 for half-times and 1 for distribution volumes [3].

Phenylephrine concentration was linked directly to mean arterial pressure (MAP) using an Emax model to describe drug effect:

$$Effect=EO+\left( \frac{Emax*Cp}{Cp+{EC}_{50}} \right)$$

Where E0 is the MAP before administration of phenylephrine, Emax is the maximum blood pressure effect, Cp (mcg.L^-1^) is the plasma phenylephrine concentration and EC_50_ (mcg.L^-1^) is the concentration producing 50% Emax.

**Quality of fit**

The quality of fit of the models to the data was sought by NONMEM’s objective function and by visual examination of plots of observed versus predicted concentrations. Models were nested and an improvement in the objective function was referred to the Chi-squared distribution to assess significance e.g. an objective function change (OBJ) of 3.84 is significant at α=0.05. Bootstrap methods provided a means to evaluate parameter uncertainty [4]. A total of 1000 replications were used to estimate parameter confidence intervals. A visual predictive check (VPC) [5], a modeling tool that estimates the concentration prediction intervals and graphically superimposes these intervals on observed concentrations after a standardized dose, was used to evaluate how well the model predicted the distribution of observed plasma concentrations. Simulation was performed using 1000 subjects with characteristics taken from subjects. For data such as these where covariates such as dose and weight are different for each patient, we used a prediction corrected VPC (PC-VPC) [6].

**References**

1. Holford NHG (1996) A size standard for pharmacokinetics. Clin Pharmacokinet 30:329-332.

2. Anderson BJ, Meakin GH (2002) Scaling for size: some implications for paediatric anaesthesia dosing. Paediatr Anaesth 12 (3):205-219.

3. Anderson BJ, Holford NH (2008) Mechanism-based concepts of size and maturity in pharmacokinetics. Annu Rev Pharmacol Toxicol 48:303-332.

4. Efron B (1979) Bootstrap methods: another look at the jackknife. Ann Statist 7 (1):1-26.

5. Post TM, Freijer JI, Ploeger BA, Danhof M (2008) Extensions to the visual predictive check to facilitate model performance evaluation. J Pharmacokinet Pharmacodyn 35 (2):185-202.

6. Karlsson MO, Savic RM (2007) Diagnosing model diagnostics. Clin Pharmacol Ther 82 (1):17-20.
